# Evaluating Lifestyle and Educational Factors Influencing Public Knowledge, Attitudes, and Practices Toward *Helicobacter pylori*‐Induced Gastric Ulcer: A Cross‐Sectional Study

**DOI:** 10.1002/hsr2.72286

**Published:** 2026-04-06

**Authors:** Asma Ghulam Mustafa, Adeel Aslam, Muhammad Aamir, Shazia Jamshed, Sumera Saeed Akhtar

**Affiliations:** ^1^ Faculty of Pharmacy University of Lahore Lahore Punjab Pakistan; ^2^ Department of Pharmacy Practice and Clinical Pharmacy, Faculty of Pharmacy Universiti Teknologi MARA, Campus Puncak Alam Puncak Alam Selangor Malaysia; ^3^ International Medical University Kuala Lumpur Malaysia; ^4^ Department of Primary Health Care University of Otago Dunedin New Zealand

**Keywords:** gastric ulcer attitude, gastric ulcer knowledge, gastric ulcer practices, *H. pylori* in Pakistan, *Helicobacter pylori*

## Abstract

**Background and Aims:**

*Helicobacter pylori* (*H. pylori*)‐induced gastric ulcer is a public health challenge in Pakistan. The current study examined the knowledge, attitudes, and practices regarding *H. pylori*‐induced gastric ulcers among the general Pakistani population.

**Methods:**

A cross‐sectional study was conducted in Lahore, Pakistan's second‐largest city, involving 385 participants from the general population selected through convenience sampling. Data were collected via interview‐administered questionnaires. Statistical analyses, including descriptive statistics, *χ* ^2^ tests, and binary logistic regression, were performed using SPSS version 25.

**Results:**

Among the participants, 51.4% were male and 48.6% female, primarily 54.1% were aged 18–24 with 57.4% holding bachelor's degrees. In general Pakistani population majority have moderate knowledge of *H. pylori*‐induced gastric ulcers, with 49.1% having heard of the condition and many were unsure about its symptoms and transmission. Regarding attitude, 86.8% recognized it as a serious health issue, and only 40.0% believed they could be infected. Preventive practices such as regular hand washing (52.2%) and proper food hygiene (43.8%) were common, but lifestyle factors like smoking and diet affected knowledge and attitudes significantly. Regression analysis highlighted that individuals aged 18–24 (odds ratio (OR) = 2.592, *p* = 0.014) and students (adjusted odds ratio (ORa) = 20.849, *p* < 0.001) showed higher knowledge, while low education (ORa = 0.151, *p* = 0.004), low income (ORa = 0.230, *p* = 0.001), and smoking (ORa = 0.379, *p* = 0.026) were associated with poorer attitudes. Students (ORa = 0.130, *p* = 0.005), and unemployment (ORa = 0.173, *p* = 0.013) were linked to poorer practices.

**Conclusion:**

The results of the current study highlight significant knowledge, attitude, and practice gaps regarding *H. pylori*‐induced gastric ulcers in the general Pakistani population. Targeted educational interventions are important to address misconceptions, promote healthy behaviors, and improve management strategies for *H. pylori*‐induced gastric ulcers in Pakistan.

## Summary

- *Helicobacter pylori* is a leading cause of gastric ulcers and a significant public health concern in Pakistan, yet public awareness remains limited.
- This study shows that while most people recognize *H. pylori*‐induced gastric ulcers as serious, many lack knowledge about symptoms, transmission, and prevention. Education, occupation, and lifestyle significantly influence knowledge and attitude.
- Culturally tailored educational programs are needed to bridge knowledge and practice gaps, promoting early detection and prevention of *H. pylori*‐related complications in the Pakistani population.

## 1 Introduction

*Helicobacter pylori* (*H. pylori*), which belongs to the family Helicobacteraceae, is a gram‐negative spiral‐shaped transmissible bacteria and pathogen [1]. Previous studies have proved that there is a strong association of *H. pylori* with various upper gastrointestinal disorders like gastric ulcer, chronic gastritis, peptic ulcer disease, gastric mucosa‐associated lymphoid tissue lymphoma, and gastric cancer [2]. *H. pylori* can survive in the gastric mucosa and cause significant inflammation and tissue damage through various virulence factors ultimately leading to gastric ulcer [3]. Several modifiable risk factors involving alcohol use, poor diet, unhygienic practices, and ignorance of the mode of transmission of *H. pylori* are well understood to interact with the development of *H. pylori*‐induced gastric ulcer [4]. Evidence from prior studies highlights that the general population often has insufficient knowledge about *H. pylori* and its potential complications [5, 6, 7]. Attitudes toward *H. pylori*‐induced gastric ulcers are often suboptimal, with many individuals perceiving their lifetime risk of acquiring *H. pylori* infection or developing gastric ulcers as “about the same as” or “less than” that of their peers of the same age and gender [7, 8]. Some do not perceive themselves as being at risk of *H. pylori*‐induced gastric ulcer even in the presence of a 40% underlying prevalence of *H. pylori* in their area [9]. Generally, adopting healthy practices has been linked to lower risks of *H. pylori*‐induced gastric ulcer [10, 11]. These studies have shown that there is very limited research on *H. pylori* especially *H. pylori*‐induced gastric ulcer that can be prevented through early screening and treatment. However, currently, there is no sufficient research data available on the level of the general public's knowledge regarding the symptoms, risk factors, transmission, and prevention of *H. pylori*‐induced gastric ulcer in the Pakistani population. The main objectives of the study are to assess the level of knowledge, attitudes, and practices of the general Pakistani population toward *H. pylori*‐induced gastric ulcer.

## 2 Materials and Methods

### 2.1 Study Design

A cross‐sectional study [12] was conducted to assess the knowledge, attitudes, and practices regarding *H. pylori*‐induced gastric ulcer among the general Pakistani population.

### 2.2 Study Population

The participants in the current study consisted of the general population, aged 18 and above without any communication barriers. Individuals with apparent communication barriers (e.g., language difficulties, hearing or speech impairments) were identified through initial interaction and excluded to ensure accurate data collection. Those who did not meet the inclusion criteria mentioned above were excluded from the study. Potential participants were briefed about the study's objectives and voluntarily consented to participate by signing an informed consent form. Participation was entirely voluntary, and no incentives were provided. Individuals who declined to provide informed consent were also excluded. To maintain confidentiality and anonymity, it was stated on the consent form that no participant information would be shared with anyone, and the data collected would remain private.

### 2.3 Study Setting and Sampling Technique

Data were collected in Lahore, Pakistan's second‐largest city, at public venues including shopping malls, outside hospitals, and supermarkets. A convenience sampling (intercept) approach was used. Venues were selected to reflect different areas of Lahore and were visited on weekdays and weekends during morning/afternoon/evening time blocks. At each venue, trained investigators approached (all consecutive adults entering/exiting at fixed points) and conducted a brief eligibility screen (≥ 18 years and no apparent communication barriers). Eligible and willing individuals were enrolled after written informed consent. If an individual declined, the next eligible person was approached. To reduce duplicate participation, participants were asked whether they had previously taken part in the survey.

### 2.4 Sample Size

The sample size was calculated using Raosoft's software using the population size of Lahore (13.98 million) [13], a 95% confidence level, 5% margin of error, and a 50% response distribution [14]. The computed sample size was 384. The main focus of the current study was to collect data from the general population, with a target of 410 respondents. Out of these, 385 fully completed questionnaires were included in the study.

### 2.5 Data Collection

Data were collected through interview‐administered questionnaires to accommodate participants with varying literacy levels and to ensure clarity of responses. Prior to data collection, all investigators received standardized training on questionnaire administration to ensure consistency and minimize interviewer bias. Data collection was carried out over a period of 6 months from February to July 2024, with field investigators supervised regularly to ensure adherence to protocols.

### 2.6 Questionnaire Development

The questionnaire was developed after a thorough review of existing literature [6, 7, 15, 16, 17]. Originally, it was adapted to ensure consistency among its questions, with modifications made as necessary.

### 2.7 Reliability

Reliability of questionnaire was assessed for internal consistency and corrected item‐total correlations using Cronbach's *α* coefficient.

### 2.8 Validity

Content and face validity, as well as the Content Validity Index (CVI) and Face Validity Index (FVI), were also calculated. The face validity of questionnaire was assessed by 10 members of the general public. A panel of ten experts specialized in pharmacy practice assessed the content validity of the final version of the questionnaire.

### 2.9 Data Collection Tool

The final version of the questionnaire consisted of five sections covering various aspects. Each section comprises of closed‐ended questions. The demographic section included eight questions focusing on gender, age, educational level, occupation, monthly household income, and personal habits such as smoking, consumption of spicy or highly acidic foods, and regular use of NSAIDs. The knowledge section included five questions assessing participants' knowledge of whether they had ever heard of *H. pylori*, symptoms, risk factors, mode of transmission, and preventive measures related to *H. pylori*‐induced gastric ulcer. The third section consisted of five questions exploring attitudes toward *H. pylori*‐induced gastric ulcer and health‐seeking behavior. The fourth practices section included five questions regarding participants' behaviors concerning *H. pylori*‐induced gastric ulcer, with responses provided on a 5‐point Likert scale: Always, Most of the time, Occasionally, Rarely, and Never. Lastly, the fifth segment focused on information sources and awareness campaigns, comprising three specific questions aimed at understanding participants' perspectives on these aspects.

### 2.10 Statistical Analysis

All data were coded and entered into Microsoft Excel, then transferred to SPSS version 25 for analysis. Categorical data, such as sociodemographic characteristics, were presented as frequencies and percentages. Each correct answer scored one point, while incorrect answers scored zero. Thus, the maximum score for the knowledge section was 5, with a minimum of 0 regarding *H. pylori*‐induced gastric ulcer. For the attitude and practices section, it was the same. Knowledge, attitudes, and practices were categorized as either good or poor, with cut‐off scores defined as poor (0%–50%) and good (> 50%). *χ* ^2^ [18] and binary logistic regression analysis [19] were employed to examine the association between the participants' demographic characteristics and their knowledge, attitudes, and practices toward *H. pylori*‐induced gastric ulcer. Odds ratios (ORs) and adjusted odds ratios (ORa) with 95% confidence intervals (CI) and *p* values were reported. All statistical tests were two‐sided, and a *p* < 0.05 was considered statistically significant. Additionally, forest plots were used to visualize the interaction effects, providing a clearer insight into the impact of confounding variables on the relationships between variables. All analyses (including subgroup analyses) were prespecified in the study protocol and none were exploratory.

### 2.11 Ethical Approval

Before starting the current study, ethical approval for data collection was obtained from the Ethical Committees of the University of Lahore (IREC‐2024‐03H). All the study procedures were performed according to the Declaration of Helsinki guidelines for human research.

## 3 Results

### 3.1 Reliability and Validity

The internal consistency of the entire questionnaire is found to be satisfactory, with a Cronbach's *α* value of 0.872. Additionally, internal consistency was assessed for each section separately. For the knowledge section, Cronbach's *α* value was 0.882, and for the attitude section, it was 0.752. In the practice section, and information sources/awareness campaigns section, Cronbach's *α* value was 0.749.

The FVI and CVI of the questionnaire were also within the acceptable range. The FVI for the questionnaire was calculated to be 0.894, and the CVI for the questionnaire was calculated to be 0.922.

### 3.2 Demographic Characteristics

Overall, *n* = 385 (100.0%) fully‐completed questionnaires were received from the participants. In terms of gender distribution, approximately 51.4% of the participants were male, while 48.6% were female. Regarding age groups, the majority (54.1%) fell within the 18–24 age, followed by 23.6% in the 25–34 range. In terms of education, a significant portion (57.4%) held bachelor's degrees, with 17.7% having attained master's degrees or higher. Occupation‐wise, students constituted the largest group at 43.4%, followed by employed individuals at 20.5%. Concerning monthly household income, 28.0% fell within the 25,000–50,000 PKR, and an almost equal proportion (24.7%) fell within the 50,000–100,000 PKR. Smoking was reported by 12.7% of respondents, while 47.8% reported consuming spicy or highly acidic foods. Lastly, 38.4% indicated the use of NSAIDs (Table 1).

**Table 1:** Demographic characteristics.

Demographic characteristics	Frequency (*n* = 385)	Percentage
*Gender*		
Male	198	51.4%
Female	187	48.6%
*Age (years)*		
18–24	208	54.1%
25–34	91	23.6%
35–44	24	6.2%
45–54	30	7.8%
55 and above	32	8.3%
*Educational level*		
Primary education or below	23	6.0%
Secondary education	27	7.0%
Higher secondary education	46	11.9%
Bachelor's degree	221	57.4%
Master's degree or higher	68	17.7%
*Occupation*		
Employed	79	20.5%
Self‐employed	41	10.6%
Unemployed	78	20.3%
Student	167	43.4%
Retired	20	5.2%
*Monthly household income*		
< 25,000	85	22.1%
25,000–50,000	108	28.0%
50,000–100,000	95	24.7%
More than 100,000	97	25.2%
*Do you smoke?*		
Yes	49	12.7%
No	336	87.3%
*Do you consume spicy or highly acidic foods?*		
Yes	184	47.8%
No	201	52.2%
*Do you use NSAIDs (e.g., aspirin, ibuprofen, Panadol)?*		
Yes	148	38.4%
No	237	61.6%

### 3.3 Knowledge, Attitude, and Practices

Among the participants, 49.1% had heard of *H. pylori*‐induced gastric ulcers; however, gaps in knowledge were observed, as 41.8% were unaware of the symptoms and 41.3% were unsure about its transmission. A significant portion, 41.3%, identified spicy and contaminated food as a risk factor, but many were unaware of others. In terms of prevention, 36.2% emphasized proper food hygiene, while 35.6% did not know effective measures. Attitudes show that 86.8% view the condition as serious, yet only 40.0% believe they are at risk of infection. While 73.5% agree on the importance of early screening, 74.5% would visit a doctor if symptomatic. Practices indicate that 52.2% regularly wash hands before meals, and 43.8% adhere to food hygiene. Despite 62.3% not encountering awareness materials, 77.7% are willing to participate in campaigns, highlighting a strong interest in increasing awareness (Table 2).

**Table 2:** Knowledge, attitude, and practices.

Questions	Variables	Frequency (*n* = 385)	Percentage
*Knowledge*			
Have you ever heard of *H. pylori*‐induced gastric ulcer?	Yes	189	49.1%
	No	128	33.2%
	Not sure	68	17.7%
What are the symptoms of *H. pylori*‐induced gastric ulcer?	Abdominal pain	128	33.2%
	Nausea and vomiting	61	15.8%
	Indigestion	35	9.2%
	I don't know	161	41.8%
How *H. pylori* infection is primarily transmitted?	Contaminated food and water	161	41.8%
	Person‐to‐person contact	24	6.3%
	Poor hygiene practices	41	10.6%
	I don't know	159	41.3%
What are the risk factors of *H. pylori*‐induced gastric ulcer?	Eating spicy and contaminated food	159	41.3%
	Smoking	18	4.7%
	Alcohol consumption	13	3.4%
	Regular use of nonsteroidal anti‐inflammatory drugs	41	10.6%
	I don't know	154	40.0%
What are the preventive measures to reduce the risk of *H. pylori*‐induced gastric ulcer?	Regular hand washing	24	6.2%
	Proper food hygiene practices	139	36.2%
	Avoidance of contaminated water	46	11.9%
	Quitting smoking	21	5.5%
	Reducing alcohol consumption	9	2.3%
	None	9	2.3%
	I don't know	137	35.6%
*Attitude*			
Do you consider *H. pylori*‐induced gastric ulcer a serious health issue?	Yes	334	86.8%
	No	51	13.2%
Are you concerned about the potential risks of *H. pylori* infection, such as gastric ulcer?	Yes	295	76.6%
	No	90	23.4%
Do you think you could be infected with *H. pylori*‐induced gastric ulcer?	Yes	154	40.0%
	No	89	23.1%
	Not sure	142	36.9%
Do you consider early screening and diagnosis important in the treatment and prevention of *H. pylori*‐induced gastric ulcer?	Yes	283	73.5%
	No	39	10.1%
	Not sure	63	16.4%
If you suspect you have *H. pylori*‐induced gastric ulcer or related symptoms, what action would you take?	Visit a doctor for medical advice	287	74.5%
	Self‐medicate with over‐the‐counter drugs	42	10.9%
	Ignore the symptoms	17	4.4%
	Not sure	39	10.2%
*Practices*			
How often do you avoid drinking contaminated water to prevent *H. pylori*‐induced gastric ulcer?	Always	185	48.1%
	Most of the time	116	30.1%
	Occasionally	39	10.1%
	Rarely	22	5.7%
	Never	23	6.0%
How often do you practice regular hand washing before meals to prevent *H. pylori*‐induced gastric ulcer?	Always	201	52.2%
	Most of the time	139	36.1%
	Occasionally	26	6.8%
	Rarely	14	3.6%
	Never	5	1.3%
How often do you practice proper food hygiene practices to prevent *H. pylori*‐induced gastric ulcer?	Always	169	43.8%
	Most of the time	151	39.2%
	Occasionally	31	8.1%
	Rarely	18	4.7%
	Never	16	4.2%
How frequently do you engage in regular physical exercise to prevent *H. pylori*‐induced gastric ulcer?	Always	76	19.7%
	Most of the time	79	20.5%
	Occasionally	116	30.2%
	Rarely	89	23.1%
	Never	25	6.5%
How frequently do you consult a healthcare professional when you have digestive symptoms?	Always	71	18.4%
	Most of the time	112	29.1%
	Occasionally	86	22.3%
	Rarely	97	25.3%
	Never	19	4.9%
*Participation of the general Pakistani population in awareness campaigns*			
Have you come across any awareness campaigns or educational materials about *H. pylori*‐induced gastric ulcer and its associated risks?	Yes	145	37.7%
	No	240	62.3%
Would you be willing to participate in more awareness campaigns if they were conducted?	Yes	299	77.7%
	No	86	22.3%

### 3.4 Level of Knowledge, Attitude, and Practices

The results indicate significant disparities in the levels of knowledge, attitudes, and practices regarding *H. pylori*‐induced gastric ulcers across different demographic groups in Pakistan. Males exhibit slightly higher levels of good knowledge (31.9%) compared to females (26.5%), and the highest knowledge found among 18–24 year olds (34.5%) and those with a bachelor's degree (36.6%, *p* < 0.001). Students also showed significant knowledge (34.8%, *p* < 0.001), with higher income linked to better knowledge (18.2%, *p* = 0.003). Nonsmokers (50.2%) and those avoiding spicy foods (32.6%) also had better knowledge. In terms of attitude, males (42.1%) and 18–24 year olds (42.9%) had a higher proportion of good attitudes, with bachelor's degree holders leading at 46.8% (*p* < 0.001). Nonsmokers showed significantly better attitudes (70.4%) compared to smokers (9.4%), and those not consuming spicy foods also fare better (40.1%). Practice levels were generally low, with females slightly outperforming males (16.6% vs. 14.8%), while the 18–24 age group had the lowest good practices (12.9%, *p* = 0.004). Educational differences are marked, with bachelor's degree holders at 15.6% for good practices, but also having the highest poor practices (41.8%). Nonsmokers showed better practices (27.8%), and those not using NSAIDs had higher good practices (21.6%, *p* = 0.035). Overall, education, income, and lifestyle factors significantly influence knowledge, attitude, and practices regarding *H. pylori*‐induced gastric ulcer (Table 3).

**Table 3:** Comparison of knowledge, attitude, and practice with demographic characteristics.

Demographic characteristics	Level of knowledge (*n* = 385)		*p*	Level of attitude (*n* = 385)		*p*	Level of practices (*n* = 385)		*p*
	Good knowledge	Poor knowledge		Good attitude	Poor attitude		Good practices	Poor practices	
*Gender*									
Male	123 (31.9%)	75 (19.5%)	0.132	162 (42.1%)	36 (9.4%)	0.297	57 (14.8%)	141 (36.7%)	0.251
Female	102 (26.5%)	85 (22.1%)		145 (37.7%)	42 (10.8%)		64 (16.6%)	123 (31.9%)	
*Age*									
18–24	133 (34.5%)	75 (19.5%)	0.061	165 (42.9%)	43 (11.2%)	0.429	50 (12.9%)	158 (41.0%)	**0.004** **
25–34	48 (12.5%)	43 (11.2%)		78 (20.3%)	13 (3.4%)		35 (9.3%)	56 (14.5%)	
35–44	12 (3.1%)	12 (3.1%)		17 (4.4%)	7 (1.8%)		7 (1.8%)	17 (4.4%)	
45–54	19 (4.9%)	11 (2.9%)		23 (5.9%)	7 (1.8%)		16 (4.2%)	14 (3.6%)	
55 and above	13 (3.4%)	19 (4.9%)		24 (6.2%)	8 (2.1%)		13 (3.4%)	19 (4.9%)	
*Educational level*									
Primary education or below	6 (1.6%)	17 (4.4%)	**< 0.001** ***	9 (2.3%)	14 (3.6%)	**< 0.001** ***	6 (1.5%)	17 (4.4%)	0.110
Secondary education	8 (2.1%)	19 (4.9%)		22 (5.7%)	5 (1.3%)		9 (2.4%)	18 (4.7%)	
Higher secondary education	27 (7.0%)	19 (4.9%)		37 (9.6%)	9 (2.3%)		16 (4.2%)	30 (7.8%)	
Bachelor's degree	141 (36.6%)	80 (20.8%)		180 (46.8%)	41 (10.7%)		60 (15.6%)	161 (41.8%)	
Master's degree or higher	43 (11.2%)	25 (6.5%)		59 (15.4%)	9 (2.3%)		30 (7.8%)	38 (9.8%)	
*Occupation*									
Employed	39 (10.1%)	40 (10.4%)	**< 0.001** ***	66 (17.1%)	13 (3.4%)	0.420	32 (8.3%)	47 (12.2%)	**0.001** **
Self‐employed	19 (4.9%)	22 (5.7%)		32 (8.3%)	9 (2.3%)		17 (4.4%)	24 (6.2%)	
Unemployed	27 (7.1%)	51 (13.2%)		58 (15.1%)	20 (5.2%)		23 (5.9%)	55 (14.3%)	
Student	134 (34.8%)	33 (8.6%)		137 (35.6%)	30 (7.8%)		37 (9.6%)	130 (33.8%)	
Retired	6 (1.6%)	14 (3.6%)		14 (3.6%)	6 (1.6%)		12 (3.2%)	8 (2.1%)	
*Monthly household income*									
< 25,000 PKR	38 (9.9%)	47 (12.2%)	**0.003** **	56 (14.5%)	29 (7.5%)	**0.001** **	22 (5.7%)	63 (16.4%)	0.559
25,000–50,000 PKR	62 (16.1%)	46 (11.9%)		86 (22.3%)	22 (5.7%)		38 (9.9%)	70 (18.2%)	
50,000–100,000 PKR	55 (14.3%)	40 (10.4%)		79 (20.5%)	16 (4.3%)		29 (7.5%)	66 (17.1%)	
More than 100,000 PKR	70 (18.2%)	27 (7.0%)		86 (22.3%)	11 (2.9%)		32 (8.3%)	65 (16.9%)	
*Do you smoke?*									
Yes	32 (8.3%)	17 (4.4%)	0.297	36 (9.4%)	13 (3.4%)	0.242	14 (3.6%)	35 (9.1%)	0.389
No	193 (50.2%)	143 (37.1%)		271 (70.4%)	65 (16.8%)		107 (27.8%)	229 (59.5%)	
*Do you consume spicy or highly acidic food?*									
Yes	100 (25.9%)	84 (21.8%)	0.119	145 (37.7%)	39 (11.1%)	0.662	47 (12.3%)	137 (35.6%)	**0.011** *
No	125 (32.6%)	76 (19.7%)		162 (40.1%)	39 (11.1%)		74 (19.2%)	127 (32.9%)	
*Do you use NSAIDs regularly (e.g., aspirin, ibuprofen, Panadol)?*									
Yes	80 (20.9%)	68 (17.6%)	0.167	129 (33.5%)	19 (4.9%)	**0.004** **	38 (9.8%)	110 (28.6%)	**0.035** *
No	145 (37.6%)	92 (23.9%)		178 (46.2%)	59 (15.4%)		83 (21.6%)	154 (40%)	

*Note:* Percentages are calculated based on the total study population (*n* = 385). The sum of “Good” and “Poor” knowledge, attitude, or practice across all groups equals 100%. *p* values from *χ* ^2^ tests. Bold values indicate statistically significant results.

* *p* < 0.05;

** *p* < 0.01;

*** *p* < 0.001.

### 3.5 Regression Analysis

The regression analysis showed several significant factors influencing knowledge, attitudes, and practices regarding *H. pylori*‐induced gastric ulcers among the general Pakistani population. Males were slightly more likely to have good knowledge than females (ORa = 1.247, *p* = 0.402). Individuals aged 18–24 showed higher likelihoods of good knowledge (OR = 2.592, *p* = 0.014). Individuals with primary education or below were significantly less likely to have a good attitude than those with a Master's degree or higher, even after adjustment (ORa = 0.151, *p* = 0.004). Students demonstrated a strong likelihood of good knowledge (ORa = 20.849, *p* < 0.001). For attitude, primary education or below was linked to a significantly lower likelihood of good attitude (ORa = 0.151, *p* = 0.004), while lower income also negatively impacted attitude (ORa = 0.230, *p* = 0.001). Smoking was associated with poorer attitudes (ORa = 0.379, *p* = 0.026), but regular NSAID users were more likely to have a good attitude (ORa = 3.251, *p* = 0.001). In terms of practices, males were less likely to adopt good practices than females (ORa = 0.768, *p* = 0.307), and younger individuals [18, 19, 20, 21, 22, 23, 24] showed variability in practice likelihoods. Students were less likely to engage in poor practices (ORa = 0.130, *p* = 0.005), while lower income and certain lifestyle factors like NSAID use and diet affected practice levels. These findings emphasize the influence of education, income, occupation, and lifestyle factors on knowledge, attitudes, and practices related to gastric ulcers (Table 4).

**Table 4:** Regression Analysis.

Variable	Good knowledge				Good attitude				Good practices			
	Univariate		Multivariate		Univariate		Multivariate		Univariate		Multivariate	
	OR (95% CI)	*p*	ORa (95% CI)	*p*	OR (95% CI)	*p*	ORa (95% CI)	*p*	OR (95% CI)	*p*	ORa (95% CI)	*p*
*Gender*												
Male	1.367 (0.910–2.052)	0.132	1.247 (0.744–2.092)	0.402	1.303 (0.792–2.146)	0.297	1.258 (0.683–2.317)	0.462	0.777 (0.505–1.196)	0.251	0.768 (0.463–1.274)	0.307
Female	*Ref*		*Ref*		*Ref*		*Ref*		*Ref*		*Ref*	
*Age*												
18–24	2.592 (1.212–5.543)	**0.014** *	0.540 (0.160–1.827)	0.322	1.279 (0.537–3.046)	0.578	0.641 (0.166–2.483)	0.520	0.463 (0.213–1.003)	0.051	1.298 (0.376–4.483)	0.680
25–34	1.631 (0.721–3.692)	0.240	0.647 (0.193–2.173)	0.481	2.000 (0.741–5.396)	0.171	1.451 (0.359–5.874)	0.602	0.913 (0.401–2.079)	0.829	1.945 (0.56–6.662)	0.290
35–44	1.462 (0.503–4.247)	0.486	1.131 (0.282–4.534)	0.862	0.810 (0.246–2.660)	0.728	0.417 (0.088–1.964)	0.269	0.602 (0.195–1.859)	0.378	1.058 (0.256–4.369)	0.938
45–54	2.524 (0.906–7.031)	0.076	2.180 (0.578–8.228)	0.250	1.095 (0.342–3.509)	0.878	0.566 (0.121–2.655)	0.471	1.670 (0.611–4.568)	0.318	3.647 (0.959–13.874)	0.058
55 and above	*Ref*		*Ref*		*Ref*		*Ref*		*Ref*		*Ref*	
*Educational level*												
Primary education or below	0.205 (0.072–0.588)	**0.003** **	0.399 (0.116–1.369)	0.144	0.098 (0.033–0.292)	**< 0.001** ***	0.151 (0.042–0.537)	**0.004** **	0.447 (0.157–1.273)	0.132	0.424 (0.127–1.410)	0.162
Secondary education	0.245 (0.094–0.641)	**0.004** **	0.265 (0.088–0.794)	**0.018** *	0.671 (0.203–2.224)	0.514	0.703 (0.190–2.596)	0.597	0.633 (0.249–1.609)	0.337	0.776 (0.276–2.187)	0.632
Higher secondary education	0.826 (0.384–1.778)	0.625	0.744 (0.291–1.903)	0.537	0.627 (0.228–1.724)	0.366	0.709 (0.229–2.197)	0.551	0.676 (0.312–1.463)	0.320	0.893 (0.367–2.175)	0.803
Bachelor's degree	1.025 (0.583–1.801)	0.932	0.685 (0.334–1.404)	0.301	0.670 (0.307–1.460)	0.313	0.808 (0.335–1.947)	0.635	0.472 (0.269–0.829)	**0.009** **	0.872 (0.445–1.708)	0.689
Master's degree or higher	*Ref*		*Ref*		*Ref*		*Ref*		*Ref*		*Ref*	
*Occupation*												
Employed	2.275 (0.794–6.522)	0.126	3.256 (0.783–13.545)	0.105	2.176 (0.706–6.710)	0.176	1.487 (0.320–6.904)	0.613	0.454 (0.167–1.235)	0.122	0.271 (0.069–1.069)	0.062
Self‐employed	2.015 (0.647–6.278)	0.227	2.640 (0.584–11.942)	0.207	1.524 (0.455–5.105)	0.495	1.887 (0.371–9.609)	0.444	0.472 (0.159–1.403)	0.177	0.294 (0.068–1.275)	0.102
Unemployed	1.235 (0.426–3.580)	0.697	2.006 (0.479–8.395)	0.340	1.243 (0.421–3.671)	0.694	1.239 (0.266–5.781)	0.785	0.279 (0.101–0.772)	**0.014** *	0.173 (0.043–0.688)	**0.013** *
Student	9.475 (3.385–26.524)	**< 0.001** ***	20.849 (4.743–91.637)	**< 0.001** ***	1.957 (0.695–5.508)	0.203	1.939 (0.402–9.368)	0.410	0.190 (0.072–0.499)	**0.001** **	0.130 (0.032–0.536)	**0.005** **
Retired	*Ref*		*Ref*		*Ref*		*Ref*		*Ref*		*Ref*	
*Monthly household income*												
< 25,000 PKR	0.312 (0.168–0.578)	**< 0.001** ***	0.281 (0.132–0.597)	0.281	0.247 (0.114–0.534)	**< 0.001** ***	0.230 (0.096–0.552)	**0.001** **	0.709 (0.373–1.351)	0.296	0.738 (0.358–1.521)	0.410
25,000–50,000 PKR	0.520 (0.290–0.933)	**0.028** *	0.568 (0.281–1.151)	0.568	0.500 (0.229–1.094)	0.083	0.424 (0.179–1.003)	0.051	1.103 (0.618–1.967)	0.741	0.890 (0.465–1.707)	0.727
50,000–100,000 PKR	0.530 (0.290–0.969)	**0.039** *	0.473 (0.232–0.962)	0.473	0.632 (0.276–1.443)	0.276	0.679 (0.281–1.643)	0.391	0.893 (0.486–1.640)	0.714	0.803 (0.413–1.563)	0.519
More than 100,000 PKR	*Ref*		*Ref*		*Ref*		*Ref*		*Ref*		*Ref*	
*Do you smoke?*												
Yes	1.395 (0.745–2.610)	0.298	1.151 (0.514–2.575)	0.733	0.664 (0.333–1.324)	0.245	0.379 (0.161–0.891)	**0.026** *	0.856 (0.442–1.658)	0.645	1.045 (0.483–2.262)	0.910
No	*Ref*		*Ref*		*Ref*		*Ref*		*Ref*		*Ref*	
*Do you consume spicy or highly acidic food?*												
Yes	0.724 (0.482–1.087)	0.119	0.579 (0.343–0.978)	**0.041** *	0.895 (0.544–1.472)	0.662	0.657 (0.363–1.186)	0.163	0.589 (0.380–0.912)	**0.018** *	0.736 (0.443–1.223)	0.237
No	*Ref*		*Ref*		*Ref*		*Ref*		*Ref*		*Ref*	
*Do you use NSAIDs regularly (e.g., aspirin, ibuprofen, Panadol)?*												
Yes	0.746 (0.493–1.131)	0.168	0.895 (0.530–1.512)	0.679	2.250 (1.280–3.958)	**0.005** **	3.251 (1.673–6.317)	**0.001** **	0.641 (0.407–1.011)	0.055	0.599 (0.354–1.013)	0.056
No	*Ref*		*Ref*		*Ref*		*Ref*		*Ref*		*Ref*	

*Note: p* values from binary logistic regression. Bold values indicate statistically significant results.

* *p* < 0.05;

** *p* < 0.01;

*** *p* < 0.001.

### 3.6 Forest Plot of Regression Analysis

**Figure 1:**
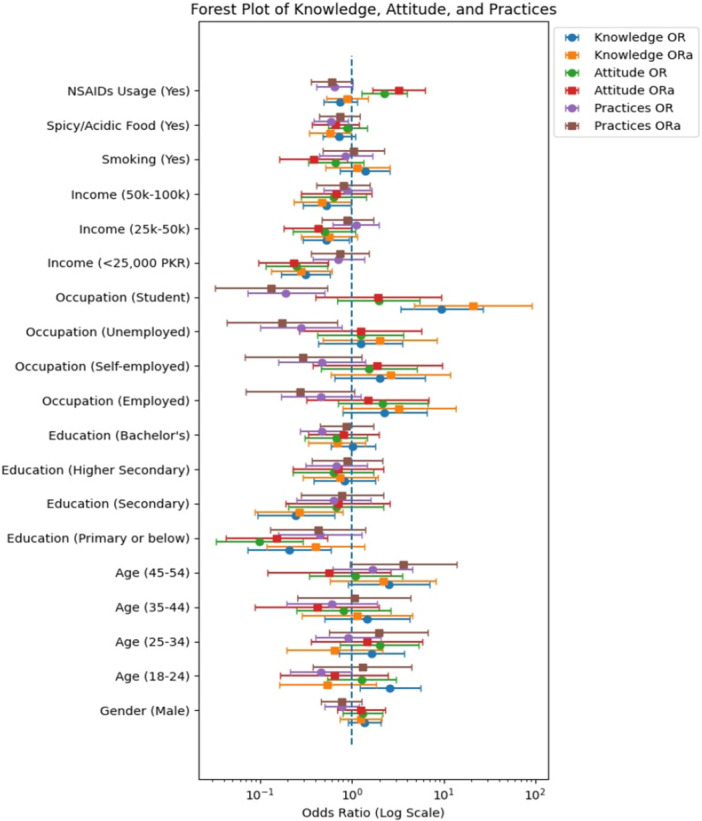
Forest plot of knowledge, attitude, and practices. Forest plot displaying odds ratio (OR) and adjusted odds ratios (ORs) with 95% confidence intervals (CIs) from the binary logistic regression analysis of factors associated with good knowledge, positive attitudes, and good practices regarding *Helicobacter pylori*‐induced gastric ulcers among the general Pakistani population (*n* = 385).

The forest plot (Figure 1) highlights key factors influencing knowledge, attitude, and practices (KAP). The *X*‐axis is on a logarithmic scale, displaying ORs where 1 (marked by a dashed line) represents no effect values to the left indicate a lower likelihood, while those to the right indicate a higher likelihood. Horizontal lines extending from each marker denote 95% CI if these lines cross the reference line at 1, the factor is statistically insignificant. Education is a strong predictor, with students having significantly higher odds of good knowledge (OR = 9.47, ORa = 20.85, *p* < 0.001), while individuals with lower education levels show reduced odds of good attitude and practices. Employment status also plays a role; unemployed individuals have lower odds of good practices (ORa = 0.173, *p* = 0.013), suggesting joblessness negatively impacts health behaviors. Smoking is linked to a poorer attitude (ORa = 0.379, *p* = 0.026), while regular NSAID use is associated with a better attitude (ORa = 3.251, *p* = 0.001). Lower household income and frequent consumption of spicy or acidic foods correlate with poorer knowledge and practices. These findings emphasize the importance of education, employment, and lifestyle choices in shaping health‐related behaviors, highlighting the need for targeted interventions.

### 3.7 Source of Information and Awareness Campaign

**Figure 2:**
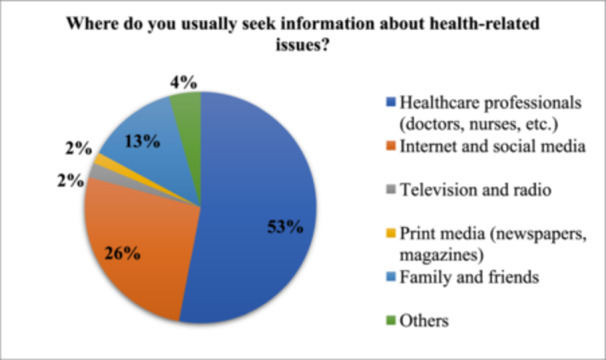
Sources of information for the general Pakistani population (*n* = 385).

A significant majority, 53%, rely on healthcare professionals like doctors and nurses. The internet and social media are also popular, with 26% turning to these platforms for health information. Family and friends are the next most common source at 13%. Traditional media like television and radio, and print media such as newspapers and magazines, are less frequently used, with 2% and 2%, respectively (Figure 2).

## 4 Discussion

To the best of our knowledge, this study is the first to explore the knowledge, attitudes, and practices of the public regarding *H. pylori*‐induced gastric ulcer in the Pakistan. The knowledge of *H. pylori*‐induced gastric ulcer in the general Pakistani population is unclear. In the current study, the majority of the participants have poor knowledge (41.6%) about *H. pylori*‐induced gastric ulcer. Improving knowledge about this condition is important, especially among those with lower education levels. This lack of knowledge is a global issue [6], as proved by previous studies that are conducted in China [9, 20], South Korea [21, 22], and North America [23, 24] also reported poor knowledge of *H. pylori*‐induced gastric ulcers. Specifically, there is limited knowledge about *H. pylori* transmission [20, 22, 24], as highlighted by the fact that only 41.8% of the participants in the current study identified contaminated food and water as a possible source of *H. pylori*‐induced gastric ulcers. Wynne and colleagues reported that only 26% of the participants identified water as a possible source of *H. pylori*‐induced gastric ulcer [24]. This emphasizes the importance of promoting water sanitation and hygienic practices to prevent waterborne diseases. Furthermore, in the current study, over 41.8% of participants were unable to identify any symptoms of gastric ulcers, a finding consistent with a study from China where over 50% of participants showed a similar lack of knowledge regarding warning signs of gastric ulcers [17]. Another research conducted among the Jordanian population showed that abdominal pain, nausea/vomiting, and abdominal bloating were correctly identified as symptoms by 75.5%, 68.4%, and 59.8% of participants, respectively [25]. In contrast, the current study findings show that participants demonstrated varying levels of knowledge regarding symptoms of gastric ulcers, with 33.2% correctly identifying abdominal pain, 15.8% recognizing nausea and vomiting, and 9.2% identifying indigestion as potential symptoms. This difference in symptom recognition may be due to differences in educational initiatives and public health campaigns across different regions. Additionally, the current study showed that 4.7% of the participants considered smoking, and 3.4% believed alcohol consumption to be risk factors for *H. pylori*‐induced gastric ulcers, aligning with findings from Oh and colleagues, where a significant portion of the population associated smoking (65.3%) and alcohol consumption (64.3%) with the development of *H. pylori*‐induced gastric ulcer [21]. These differences in risk perception could be due to variations in public health education and cultural attitudes toward these risk factors. The current research also assessed the level of knowledge among the general Pakistani population and highlighted a notable lack of knowledge about *H. pylori*‐induced gastric ulcers among the participants, particularly among individuals aged 50 years and above (4.9%) and those with a primary education level or below (4.4%). These findings are consistent with a study conducted in China, which found that individuals over 50 years old (54.8%) and those with a high school education or lower (68.6%) exhibited relatively poor knowledge regarding *H. pylori*‐induced gastric ulcers [15]. These observations suggest a global trend where higher levels of education correlate with better knowledge of *H. pylori*‐induced gastric ulcer. Interestingly, in the current study, males exhibited higher good knowledge scores (31.9%) compared to females (26.5%), although this difference was not statistically significant. This contrasts with a study conducted in the Sharjah, where females had higher average knowledge scores (58.22%) compared to males (56.25%) [7]. This variance may be attributed to the gender distribution in the current study, where males are more than females.

The attitudes of the general population toward *H. pylori*‐induced gastric ulcers play an important role in effective disease management and prevention. The majority of participants in the current study have positive attitudes toward *H. pylori*‐induced gastric ulcers, with 74.5% indicating they would seek medical help if they suspected they had such ulcers or related symptoms. This contrasts with findings from a study conducted in the Sharjah population, where only 33% expressed a willingness to seek medical assistance [7]. This may be because in this region, they prefer herbal medicines or nonmedical options, or they may be worried about the expense of healthcare. Regarding attitudes toward *H. pylori* screening, a previous study in China found that only 14% of participants believed they might be infected with *H. pylori*‐induced gastric ulcers, despite a 41% prevalence of *H. pylori* [9]. In contrast, in the current study, 40% of participants believed they could be infected with *H. pylori*‐induced gastric ulcers. This suggests that participants were aware of their self‐risk for *H. pylori*‐induced gastric ulcers. Additionally, 73.5% of the participants in the current study believed that early screening and diagnosis are important for the treatment and prevention of *H. pylori*‐induced gastric ulcers, indicating an understanding of the value of early diagnosis and screening for gastric ulcer prevention. These findings align with a study conducted in the South Korean population, where 72.1% believed that early screening and diagnosis are important for the treatment and prevention of *H. pylori*‐induced gastric ulcer [21] as well as a study in China where 83.8% believed screening is helpful for early detection of gastric ulcers [17]. These similarities may reflect cultural and dietary influences. For instance, South Korea's traditional diet, rich in fermented foods like kimchi and high salt intake, has been linked to gastric mucosal changes and has prompted public health campaigns targeting gastric cancer, indirectly improving awareness about *H. pylori* [26]. In China, widespread consumption of hot beverages, oily, and spicy foods is common, but urban–rural disparities in health education may account for uneven knowledge levels [27]. To promote regular screening, it is essential to educate the public about the importance of screening, regardless of symptoms.

The practices adopted by the general population are also critical in managing and preventing *H. pylori*‐induced gastric ulcers. The majority of participants (68.6%) in the current study demonstrated overall poor practices. Contrastingly, a study reported good practices in 74.5% of the population [28]. Handwashing before preparing meals is commonly recognized as a beneficial practice for reducing the risk of *H. pylori*‐induced gastric ulcer [29]. However, in the current study, only 48.1% of participants always avoid drinking contaminated water to prevent *H. pylori*‐induced gastric ulcers, despite its high prevalence (more than 58%) [30]. Regarding gender differences, females exhibited higher good practice scores (16.6%) compared to males (14.8%), consistent with the findings of Malek and colleagues, where females had higher good practice scores (81.28%) compared to males (72.72%) [7]. These findings highlight the need for educational programs and public health interventions to address specific gaps in knowledge, attitudes, and practices regarding *H. pylori*‐induced gastric ulcers.

### 4.1 Limitation

This study provides insights into public knowledge, attitudes, and practices regarding *H. pylori*‐induced gastric ulcers among an urban Pakistani population; however, some limitations should be considered. First, the cross‐sectional design limits causal inference because associations were assessed at a single point in time. Second, the use of convenience sampling in public venues may have introduced selection bias by disproportionately recruiting individuals who were more accessible and willing to participate, potentially overrepresenting younger, more educated urban residents and underrepresenting older adults and rural populations. Therefore, the findings may not be fully generalizable to the broader Pakistani population. Third, although multivariable regression adjusted for key sociodemographic characteristics (e.g., education, income, occupation), other contextual determinants such as urban–rural residence, access to healthcare, and regional disparities were not comprehensively captured and may influence knowledge, attitudes, and practices. Future studies using probability‐based sampling and longitudinal designs are recommended to improve representativeness and assess changes in knowledge, attitudes, and practices over time.

### 4.2 Policy Implications

The findings of this study underscore the need for targeted public health strategies to address gaps in knowledge, attitudes, and practices regarding *H. pylori*‐induced gastric ulcers in Pakistan. Integrating *H. pylori* awareness into primary healthcare training can empower healthcare workers to educate communities about prevention, early detection, and treatment. Aligning these efforts with Pakistan's National Health Strategy and initiatives such as the 2021–2025 Digestive Disease Prevention Plan could strengthen community‐based interventions. Additionally, culturally tailored health education campaigns focusing on hygiene, dietary habits, and risk factor modification should be prioritized to reduce the burden of *H. pylori*‐related complications. These approaches will support the development of sustainable prevention and management programs within Pakistan's healthcare system.

### 4.3 Future Research Directions

Future studies should adopt longitudinal designs to monitor changes in knowledge, attitudes, and practices over time, allowing for the evaluation of the long‐term effectiveness of educational interventions. Additionally, exploring omics‐level mechanistic research, such as genomics and microbiome profiling, could provide deeper insights into host–pathogen interactions and population‐specific vulnerabilities to *H. pylori*‐induced gastric ulcers. Such approaches would enhance the scientific understanding of disease progression and support the development of precision public health strategies tailored to the Pakistani population.

## 5 Conclusion

The study highlights significant knowledge, attitude, and practice gaps regarding *H. pylori*‐induced gastric ulcers among the general Pakistani population. While there is recognition of *H. pylori*‐induced gastric ulcers as a serious health concern, many lack knowledge about *H. pylori* and its associated risks. Higher education levels correlate with better knowledge, indicating the importance of education. Socioeconomic factors like occupation and income also influence health literacy. Lifestyle habits such as smoking and NSAID use impact attitudes and practices. Healthcare professionals play a crucial role in providing information, but there is a need for targeted interventions to address misconceptions and promote healthy practices. Awareness campaigns are limited, but there is a willingness to participate in future initiatives. Overall, there is an urgent need for educational programs to enhance knowledge and promote healthier attitudes and practices in preventing *H. pylori*‐induced gastric ulcers among the Pakistani population.

## Author Contributions

**Asma Ghulam Mustafa:** writing – review and editing, writing – original draft, visualization, methodology, conceptualization, investigation, validation, formal analysis. **Adeel Aslam:** conceptualization, writing – original draft, writing – review and editing, supervision. **Muhammad Aamir:** writing – review and editing, writing – original draft, conceptualization, supervision. **Shazia Jamshed:** project administration, writing – review and editing, writing – original draft, investigation. **Sumera Saeed Akhtar:** writing – original draft, writing – review and editing, formal analysis, methodology. All authors have read and approved the final version of the manuscript.

## Funding

The authors have nothing to report.

## Ethics Statement

Ethical approval for data collection was obtained from the Ethical Committees of the University of Lahore (IREC‐2024‐03H). All the study procedures were performed according to the Declaration of Helsinki guidelines for human research.

## Consent

Participants were briefed about the study's objectives and voluntarily consented to participate by signing an informed consent form.

## Conflicts of Interest

Shazia Jamshed is an editorial board member of *Health Science Reports* and a co‐author of this article. To minimize bias, she was excluded from all editorial decision‐making related to the acceptance of this article for publication. The remaining authors declare no conflicts of interest.

## 1 Transparency Statement

The lead author Sumera Saeed Akhtar affirms that this manuscript is an honest, accurate, and transparent account of the study being reported; that no important aspects of the study have been omitted; and that any discrepancies from the study as planned (and, if relevant, registered) have been explained.

## Data Availability

The data underlying this article will be shared at a reasonable request by the corresponding author. Dr. Sumera Saeed Akhtar had full access to all of the data in this study and takes complete responsibility for the integrity of the data and the accuracy of the data analysis.
